# Wnt-Frizzled Signaling Regulates Activity-Mediated Synapse Formation

**DOI:** 10.3389/fnmol.2021.683035

**Published:** 2021-06-14

**Authors:** Samuel Teo, Patricia C. Salinas

**Affiliations:** Department of Cell and Developmental Biology, University College London, London, United Kingdom

**Keywords:** Wnt, frizzled, synapse formation, neuronal activity, development

## Abstract

The formation of synapses is a tightly regulated process that requires the coordinated assembly of the presynaptic and postsynaptic sides. Defects in synaptogenesis during development or in the adult can lead to neurodevelopmental disorders, neurological disorders, and neurodegenerative diseases. In order to develop therapeutic approaches for these neurological conditions, we must first understand the molecular mechanisms that regulate synapse formation. The Wnt family of secreted glycoproteins are key regulators of synapse formation in different model systems from invertebrates to mammals. In this review, we will discuss the role of Wnt signaling in the formation of excitatory synapses in the mammalian brain by focusing on Wnt7a and Wnt5a, two Wnt ligands that play an *in vivo* role in this process. We will also discuss how changes in neuronal activity modulate the expression and/or release of Wnts, resulting in changes in the localization of surface levels of Frizzled, key Wnt receptors, at the synapse. Thus, changes in neuronal activity influence the magnitude of Wnt signaling, which in turn contributes to activity-mediated synapse formation.

## Introduction

In the central nervous system, synapses permit the rapid transmission of information from one neuron to another in a unidirectional manner. Synaptic connectivity is required for all brain functions from cognition and memory to sensory processing and motor function (Mayford et al., 2012; Wyart, 2018). The assembly of functional synapses is a complex process and its dysregulation can lead to neurodevelopmental disorders such as intellectual disability, autism spectrum disorders, and attention deficit hyperactivity disorder (Zoghbi and Bear, 2012; del Pino et al., 2018). Synapse dysfunction and loss are also key features of neurodegenerative diseases such as Alzheimer’s and Parkinson’s (Ghiglieri et al., 2018; Merluzzi et al., 2018; Rajendran and Paolicelli, 2018). The ability to promote the formation of new synapses is a potential therapeutic approach for both neurodevelopmental and neurodegenerative disorders (Lu et al., 2013). Therefore, understanding the molecular mechanisms that regulate synapse formation is a crucial step in the development of treatments for these neurological conditions.

Synapse formation requires coordinated, bidirectional signaling between the presynaptic and postsynaptic compartments (Waites et al., 2005; Südhof, 2018). The assembly of synapses is first triggered when axons reach their targets. Thereafter, secreted molecules such as brain-derived neurotrophic factor (BDNF), fibroblast growth factors (FGFs), and Wnts instruct the assembly of synapses (Lu et al., 2008; Shen and Cowan, 2010; Salinas, 2012). Synaptic adhesion molecules such as neuroligins, which bind to neurexins, their presynaptic partners, also perform synaptic organizing roles after their recruitment to the nascent synapse (Tsetsenis et al., 2014; Südhof, 2017). In addition, neuronal activity is a key regulator of synapse formation and remodeling. For example, neuronal activity regulates the trafficking of synaptic cargo including synaptic vesicles and neurotransmitter receptors to the synapse (Maas et al., 2009; Schlager and Hoogenraad, 2009). Neuronal activity also regulates the expression of genes required for synapse assembly and modulates the expression and release of synaptogenic proteins such as BDNF (West and Greenberg, 2011; Sleiman et al., 2016; Mitre et al., 2017). Thus, synapse formation is a complex process that requires different synaptic organizers and neuronal activity.

## Wnts Are Signaling Molecules That Regulate Synapse Formation

Wnts are a family of secreted glycoproteins, which act both as long–range signaling molecules through graded distribution across the tissue, as well as short–range signaling molecules that act locally on neighboring cells in direct contact with the source of Wnts or in an autocrine manner (Zecca et al., 1996; Farin et al., 2016; Loh et al., 2016). Wnts can signal through many receptors such as the Ror and Ryk tyrosine kinases (Green et al., 2014; Cerpa et al., 2015; McQuate et al., 2017). However, the best characterized Wnt signaling pathways require Frizzled (Fz) receptors, seven-transmembrane proteins containing a cysteine-rich domain (CRD) in their extracellular N-terminus for Wnt binding (Dann et al., 2001). For a more comprehensive understanding of the Wnt-Fz signaling pathways, we refer the reader to reviews on this topic such as Clevers (2006) and Gordon and Nusse (2006).

**Figure 1:**
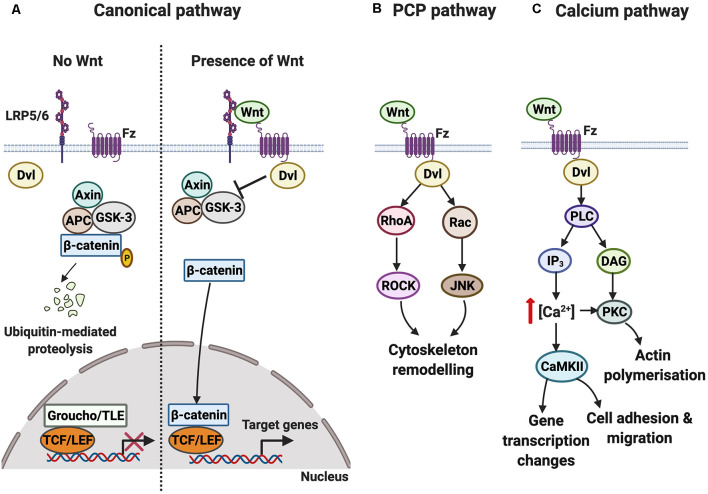
Overview of the Wnt-Fz signaling cascades. **(A)** In the canonical pathway, the protein degradation complex consisting of Axin, glycogen synthase kinase-3 (GSK-3), and Adenomatous Polyposis Coli (APC) is active when Wnt is absent. GSK-3 phosphorylates β-catenin, resulting in its degradation. Wnt target genes are not transcribed since the DNA transcription factor T-cell factor/lymphoid enhancer factor (TCF/LEF) is bound to corepressor protein Groucho/transducing-like enhancer of split (TLE). In contrast to when Wnt is present, Wnt signals through the Frizzled (Fz) receptor and its co-receptor low-density lipoprotein receptor-related protein (LRP) 5/6 to activate the cytoplasmic protein Dishevelled (Dvl), which functions as a scaffold to regulate local Wnt signaling. Activation of the pathway disrupts the protein degradation complex. β-catenin subsequently translocates into the nucleus and binds to TCF/LEF, increasing the transcription of Wnt target genes. **(B)** In the planar cell polarity (PCP) pathway, Wnt-Fz signaling through Dvl leads to the activation of RhoA and Rac. The subsequent activation of ROCK and Jun N-terminal kinase (JNK) promotes changes in the cytoskeleton. **(C)** In the calcium pathway, Wnt-Fz signaling through Dvl activates phospholipase C (PLC), increasing the levels of inositol triphosphate (IP_3_) and diacyl glycerol (DAG). This results in an increase in intracellular calcium concentration and activation of the calcium-sensitive effectors protein kinase C (PKC) and calcium/calmodulin-dependent protein kinase II (CaMKII), triggering changes in gene transcription, cell movement, and actin polymerization.

Three main Wnt signaling cascades [canonical, planar cell polarity (PCP), and calcium] can be activated by the binding of Wnts to Fz receptors. A summary of the three signaling cascades is shown in Figure 1. Of note is that only the canonical pathway, but not the PCP or calcium pathways, requires the single-pass transmembrane low-density lipoprotein receptor-related protein (LRP) 5 or 6 co-receptor, encoded by two different genes *LRP5* and *LRP6*, respectively (MacDonald and He, 2012). Activation of the three Wnt-Fz signaling pathways also requires the cytoplasmic scaffold protein Dishevelled (Dvl), which increases the efficiency of signal transduction and brings together different components of the signaling pathway (Wallingford and Habas, 2005; Sharma et al., 2018). Collectively, Wnt signaling regulates diverse aspects of neural development such as neuronal differentiation, neural stem cell proliferation, axon guidance, dendritogenesis, and synaptogenesis (Bovolenta et al., 2006; Salinas, 2012; Inestrosa and Varela-Nallar, 2015).

In this review, we will examine the contribution of Wnt-Fz signaling to synaptogenesis, particularly during postnatal development when the majority of synapses are assembled. We will also analyze the role of Wnt signaling in activity-mediated synapse formation. We specifically focus our attention on the mechanisms controlling excitatory synapse formation in the mammalian central nervous system as their assembly has been extensively characterized. Excitatory synapses are asymmetric structures consisting of presynaptic boutons that make primary contact onto dendritic spines, actin-rich postsynaptic specializations (Amaral and Pozzo-Miller, 2009). We will cover the role of Wnt signaling in excitatory synapse formation by focusing on Wnt7a and Wnt5a, as the role of these Wnt ligands in synapse formation has been demonstrated *in vivo*.

## Wnts Regulate Synapse Formation During Development

### Wnt7a Is Necessary for Presynaptic Assembly During the Early Postnatal Stages

Wnt7a plays a role in presynaptic assembly in the postnatal cerebellum. Wnt7a is expressed in granule cells in the cerebellum from postnatal day 6 (P6) and its expression increases until P22, coinciding with the peak of synapse formation between granule cells and mossy fibers, the presynaptic partners of granule cells (Lucas and Salinas, 1997). The first evidence that Wnt7a is involved in presynaptic assembly came from the observation that exposure of granule cells to Wnt7a induces axonal remodeling and accumulation of presynaptic protein clusters such as synapsin I (Lucas and Salinas, 1997). To test if Wnt7a has a role in presynaptic assembly at mossy fibers, further *in vitro* gain-of-function studies were performed. These studies showed that axonal remodeling in mossy fibers is mimicked by granule cell-conditioned medium but this activity is abolished in the presence of secreted frizzled-related protein-1 (sFRP-1), which antagonizes Wnt ligands (Hall et al., 2000). Together, these results suggest that Wnt7a is released from granule cells and acts retrogradely on mossy fiber axons to promote presynaptic assembly.

*In vivo* loss-of-function studies showed that Wnt7a regulates the formation of complex glomerular rosettes in the cerebellum. The cerebellar glomerular rosette is a multiple synaptic structure that forms between a mossy fiber axon and several dendrites of different granule cell neurons during postnatal development (Hámori and Somogyi, 1983). Analyses of *Wnt7a* null mutant mice revealed less complex glomerular rosettes at P8, consistent with defects in axon remodeling (Hall et al., 2000). In addition, the accumulation of synapsin I at glomerular rosettes of these mice is reduced (Hall et al., 2000; Ahmad-Annuar et al., 2006). Thus, Wnt7a is required for presynaptic assembly in the cerebellum, released from granule cell neurons to act retrogradely on mossy fiber axons.

Wnt7a is necessary for presynaptic protein accumulation at glomerular rosettes in the cerebellum, but mainly during the early postnatal stages. The level of synapsin I is reduced at the glomerular rosettes of *Wnt7a* mutant mice at P8 but not at P15, coinciding with an increase in the level of Wnt7b expression in granule cells (Hall et al., 2000). These results suggest that loss of Wnt7a function delays the maturation of glomerular rosettes and that Wnt7b might compensate for the loss of Wnt7a function. Although the *in vivo* role of Wnt7b in the presynaptic assembly has not been reported, *in vitro* gain-of-function studies have demonstrated that Wnt7b, like Wnt7a, increases the assembly of presynaptic boutons in mossy fibers (Ahmad-Annuar et al., 2006). To further investigate the compensatory effect of Wnt7b on the loss of Wnt7a function, *Wnt7a/Dvl1* double mutant mice were analyzed. As Dvl is involved in all Wnt signaling cascades, *Wnt7a/Dvl1* double mutant mice exhibit a stronger reduction in the accumulation of synapsin I at glomerular rosettes at P10 than *Wnt7a* or *Dvl1* single mutants (Ahmad-Annuar et al., 2006). Of interest is that the reduction in synapsin I extends beyond P15. Therefore, whilst the presence of Wnt7b might be sufficient to compensate for the loss of Wnt7a function, it is insufficient to compensate for the loss of both Wnt7a and Dvl1 function. Future loss-of-function Wnt7b studies will unravel its role in presynaptic assembly and remodeling in the cerebellum.

What pathway does Wnt7a trigger to promote presynaptic assembly? Wnt7a regulates presynaptic assembly by activating the canonical Wnt pathway. Inhibition of glycogen synthase kinase-3 (GSK-3), a key event in the activation of the canonical pathway, by lithium chloride mimics the effects of Wnt7a by inducing axonal remodeling and the accumulation of presynaptic proteins in cerebellar cultures (Lucas and Salinas, 1997; Hall et al., 2000). The involvement of the canonical pathway at the receptor level was later confirmed by Dickins (2012) in cultured hippocampal neurons, where Wnt7a also plays a role in presynaptic assembly. Dickkopf-related protein-1 (Dkk-1), a secreted Wnt antagonist that blocks the LRP5/6 receptor, attenuates the effect of Wnt7a/b on cultured hippocampal neurons. Importantly, Dkk-1 also reduces the number of synaptic vesicle clusters, suggesting that endogenous Wnts present in hippocampal neuronal cultures activate the canonical pathway to promote presynaptic assembly (Dickins, 2012).

Studies suggest that Wnt7a promotes presynaptic assembly through a divergent canonical pathway. Although inhibition of GSK-3 mimics the effect of Wnts on presynaptic assembly, inhibition of transcription with actinomycin-D does not affect the Wnt-induced increase in presynaptic protein clustering (Dickins, 2012). Therefore, Wnt7a does not promote presynaptic assembly through the classical canonical pathway that regulates transcription (Figure 1A). We propose that Wnt7a does so through a divergent canonical pathway upstream of β-catenin. Preliminary evidence suggests that the divergent canonical pathway might involve stabilization of microtubule dynamics by Wnt/GSK-3 signaling (Ciani et al., 2004). Future studies are required to characterize the role of the divergent Wnt canonical pathway during presynaptic assembly.

Which Fz receptor does Wnt7a signal through to promote presynaptic assembly? Fz5 has been identified as the relevant receptor (Sahores et al., 2010). In the postnatal mouse hippocampus, the level of Fz5 protein increases during postnatal development until P27. Additionally, Fz5 loss-of-function in hippocampal neurons using small hairpin RNA (shRNA) or by exposure to the soluble CRD domain of Fz5 (Fz5 CRD), which blocks Wnts that bind to Fz5, decreases the number of presynaptic synapsin I and bassoon puncta. Importantly, Fz5 loss-of-function blocks the synaptogenic activity of Wnt7a (Sahores et al., 2010). These results show that Fz5 is a receptor for Wnt7a and that endogenous Wnt7a-Fz5 signaling is required for the assembly of presynaptic boutons in hippocampal neurons.

### Wnt7a Is Required for Dendritic Spine Formation and Growth

In addition to its role in presynaptic assembly, Wnt7a is also required for postsynaptic assembly. To analyze the *in vivo* role of Wnt7a in dendritic spine development, the *Wnt7a/Dvl1* double mutant was used as it exhibits stronger synaptic defects than the *Wnt7a* single mutant. In the hippocampus, both dendritic spine density and spine head size are reduced in the *Wnt7a/Dvl1* double mutant (Ciani et al., 2011). These results demonstrate a requirement for Wnt7a-Dvl1 signaling in dendritic spine formation and growth in the hippocampus.

How does Wnt7a regulate postsynaptic assembly? The potent and specific Wnt antagonist Dkk-1 abolishes the Wnt7a-mediated increase in dendritic spine density in cultured hippocampal neurons, indicating that the effect of Wnt7a on dendritic spine formation is regulated by canonical signaling (Ramos-Fernández et al., 2019). In contrast, the Wnt7a-mediated increase in dendritic spine size is regulated by the calcium Wnt signaling pathway. Wnt7a increases the levels of active CaMKII phosphorylated at threonine 286 (Ciani et al., 2011). Interestingly, increased levels of phosphorylated CaMKII occur rapidly (within 2 min). Moreover, inhibition of CaMKII blocks the effect of Wnt7a on dendritic spine growth (Ciani et al., 2011). Expression of a local readout of CaMKII activity (containing the postsynaptic density protein 95 (PSD-95)-Vimentin-cyan fluorescent protein) revealed that Wnt7a activates CaMKII specifically at small dendritic spines (Ciani et al., 2011). Taken together these results suggest Wnt7a signals locally at dendritic spines to activate CaMKII and to promote dendritic spine growth. Future studies will elucidate how the canonical and calcium Wnt signaling pathways are activated to elicit the distinct processes of dendritic spine formation and their growth.

**Figure 2:**
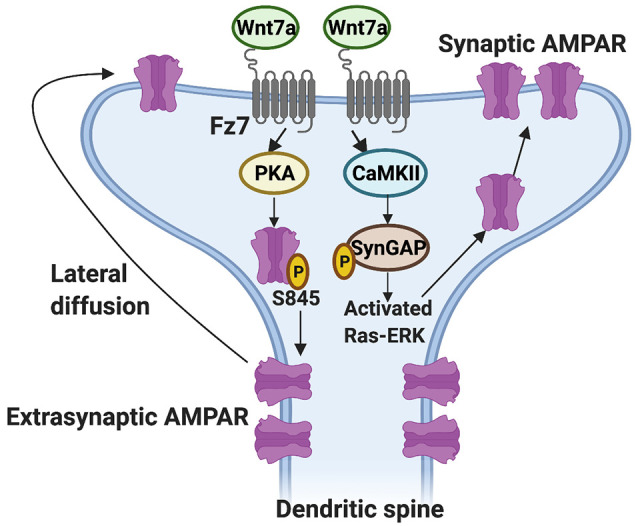
Regulation of the synaptic localization of AMPARs by Wnt7a-Fz7 signaling. Wnt7a binds to the Frizzled 7 (Fz7) receptor present at dendritic spines and activates PKA, resulting in an increase in extrasynaptic AMPARs through phosphorylation of the GluA1 subunit of AMPARs at serine (S) 845. Wnt7a might also regulate the subsequent movement of extrasynaptic AMPARs to the synapse by lateral diffusion. In addition, Wnt7a-Fz7 signaling activates calcium/calmodulin-dependent protein kinase II (CaMKII), which phosphorylates synaptic Ras GTPase-activating protein (SynGAP) and activates Ras-ERK signaling, resulting in increased levels of AMPARs at dendritic spines.

Which Wnt receptor functions on the postsynaptic side? Studies showed that Fz7 is a receptor for Wnt7a and it localizes to dendritic spines (McLeod et al., 2018). Importantly, Fz7 knockdown blocks the ability of Wnt7a to promote dendritic spine growth and synaptic strength (McLeod et al., 2018). The growth of dendritic spines is closely followed by an increase in the level of α-amino-3-hydroxy-5-methyl-4-isoxazole propionic acid receptors (AMPARs) at dendritic spines (Matsuzaki et al., 2001; Huganir and Nicoll, 2013). AMPARs are glutamate receptors crucial for synaptic connectivity and an increase in their levels enhances synaptic strength (Kessels and Malinow, 2009; Diering and Huganir, 2018). Gain- and loss-of-function studies have demonstrated that Wnt7a through Fz7 is required for the incorporation of AMPARs at dendritic spines through at least two mechanisms (McLeod et al., 2018; Figure 2). Firstly, Wnt7a-Fz7 signaling activates protein kinase A (PKA), which increases the surface levels of AMPARs at extrasynaptic sites by promoting the phosphorylation of the GluA1 subunit of AMPARs at serine-845 (S845). Consistently, the Wnt7a-mediated increase in extrasynaptic surface AMPARs is blocked by both Fz7 shRNA and PKI amide, an inhibitor of PKA. In addition, quantum dot analyses of the GluA1 subunit of AMPARs show that Wnt7a increases the fraction of immobile synaptic GluA1 and at the same time increases the diffusion of extrasynaptic GluA1, suggesting Wnt7a also regulates the lateral movement of extrasynaptic AMPARs to the synapse (McLeod et al., 2018). Secondly, Wnt7a-Fz7 signaling increases synaptic recruitment of AMPARs through the activation of CaMKII. It is known that CaMKII phosphorylates proteins such as synaptic Ras GTPase-activating protein (SynGAP) at the postsynaptic density, resulting in a reduction of SynGAP at dendritic spines and the activation of Ras-ERK signaling, thereby promoting synaptic AMPAR localization (Araki et al., 2015). Consistently, Wnt7a decreases the level of SynGAP at dendritic spines, and this effect is blocked by inhibition of CaMKII (McLeod et al., 2018). Loss-of-function of Fz7 using shRNA also blocks the long-term potentiation (LTP)-induced increase in AMPARs at dendritic spines, in agreement with the view that Wnt7a signals through Fz7 to regulate AMPARs (McLeod et al., 2018). These findings demonstrate that Wnt7a-Fz7 signaling is required for dendritic spine growth and synaptic strength by regulating the synaptic recruitment of AMPARs. These studies also revealed that Wnt7a-Fz7 signaling targets known synaptic plasticity pathways that contribute to LTP.

### Wnt5a Is Sufficient but Not Required for Dendritic Spine Formation

Wnt5a, like Wnt7a, regulates synapse formation during development at the presynaptic and postsynaptic sides. However, our understanding of the role of Wnt5a in presynaptic assembly is limited because most results are based on *in vitro* gain-of-function studies (Farías et al., 2009; Paganoni et al., 2010). In contrast, the contribution of Wnt5a to postsynaptic assembly is relatively well established. Exposure of cultured hippocampal neurons to exogenous Wnt5a promotes postsynaptic assembly by inducing the formation of dendritic spines (Varela-Nallar et al., 2010; Ramírez et al., 2016).

What is the receptor mediating the postsynaptic effects of Wnt5a? Studies suggest that Fz9 is a potential Wnt5a receptor (Ramírez et al., 2016). In synaptosomes isolated from adult rat brains, Fz9 is highly enriched in the PSD-95-containing fraction but not in the synaptophysin-enriched fraction, suggesting Fz9 is localized postsynaptically. Interestingly, Wnt5a is also coimmunoprecipitated with the CRD domain of Fz9. Importantly, Fz9 shRNA or blockade with the soluble CRD domain of Fz9 attenuates the Wnt5a-mediated increase in dendritic spine density (Ramírez et al., 2016). Thus, Wnt5a promotes dendritic spine formation, possibly through Fz9.

In contrast to the *in vitro* studies described above, *in vivo* studies showed that Wn5a is required for the maintenance of dendritic spines but not for their formation. To examine the *in vivo* role of Wnt5a in dendritic spine development and maintenance, a Wnt5a conditional knockout mice model was generated, whereby the Wnt5a locus was floxed by the pan-neuronal Nestin-Cre line expressed from embryonic day 10 (Chen et al., 2017). Wnt5a deficient mice do not exhibit defects in dendritic spine formation during postnatal development and up to 3 months of age. In contrast at 4.5 months, these mice develop defects in dendritic spine density in the hippocampus, without overt neuronal loss or change in axonal morphology. This phenotype becomes progressively more severe with age. Analyses of the downstream cascade showed that levels of the PCP pathway (phosphorylated Jun N-terminal kinase and active Ras-related C3 botulinum toxin substrate) and calcium cascade (phosphorylated CaMKII) are reduced in 3-month-old *Wnt5a* mutant mice even before the appearance of structural abnormalities. In contrast, the canonical signaling pathway evaluated by the levels of nuclear β-catenin is unchanged (Chen et al., 2017). The signaling defects are rescued by injection of adeno-associated virus (AAV)-expressing Wnt5a into the hippocampus of 3-month-old *Wnt5a* mutant mice. Furthermore, the reduction in dendritic spine number in 6-month-old *Wnt5a* mutants is reversed after 3 months of AAV-Wnt5a administration, suggesting that exogenous Wnt5a leads to the formation of new dendritic spines and the maintenance of existing ones. In summary, these results demonstrate that Wnt5a is not required for dendritic spine formation during development, possibly due to compensation by other Wnts including Wnt7a. However, Wnt5a is required for the maintenance of dendritic spines in the adult brain, likely through the PCP and/or calcium signaling pathways (Chen et al., 2017).

## Neuronal Activity Modulates The Levels of Wnts and The Localization of Their Receptors to Promote Activity-Mediated Synapse Formation

Neuronal activity, which is crucial for synapse formation and modulation of neuronal connectivity, collaborates with Wnt signaling to promote synapse formation. *In vivo* studies have shown that neuronal activity modulates the expression of Wnt7a/b in the hippocampus. Mice housed under enriched environment (EE) conditions for 3 weeks exhibit increased levels of Wnt7a/b protein in the soma and dendrites of CA3 pyramidal neurons (Gogolla et al., 2009). Mice exposed to EE also exhibit an increased number of synapses in the hippocampal stratum lucidum and higher synapse densities at mossy fiber terminals projecting onto pyramidal neurons. The synaptic changes are likely mediated by Wnt7a since these changes are abolished in the presence of the Wnt antagonist sFRP-1, whilst local injection of Wnt7a protein into the hippocampus not exposed to EE conditions induces the same synaptic changes as observed following EE (Gogolla et al., 2009). Thus, changes in neuronal activity promote an increase in Wnt7a/b levels in hippocampal neurons, leading to synaptic remodeling between mossy fiber axons and CA3 dendrites.

Wnts and neuronal activity also cooperate to promote the formation of multi-innervated spines (MIS), dendritic spines that receive multiple presynaptic inputs. Although the number of MIS is low under basal conditions, LTP significantly increases the number of these structures (Nikonenko et al., 2003). However, the mechanisms controlling the formation of MIS remain poorly understood. A recent study showed that inhibition of Wnt signaling with sFRP-3 blocks the formation of MIS induced by N-Methyl-D-aspartate-mediated chemical LTP (cLTP; McLeod et al., 2020). Importantly, exposure to Wnt7a induces MIS formation in hippocampal neurons. Hence, Wnts, possibly Wnt7a, are required for activity-mediated MIS formation in hippocampal neurons.

Neuronal activity also modulates Wnt7a/b protein levels in hippocampal neurons. Indeed, both glutamate-induced depolarization and glycine-mediated cLTP increase Wnt7a/b protein levels in cultured rat hippocampal neurons (Tabatadze et al., 2014; McLeod et al., 2018). High-frequency stimulation (HFS) in brain slices also increases Wnt7a/b protein levels in the stratum radiatum of the CA1 region (McLeod et al., 2018). Further studies showed a rapid increase in Wnt7a/b protein at dendritic spines within 5 min of LTP induction. The fast accumulation of Wnt7a/b protein suggests that neuronal activity modulates the recruitment of Wnt7a/b protein to neuronal processes or promotes its release (McLeod et al., 2018). In contrast to Wnt7a, neither cLTP nor blockade of neuronal activity with L-type calcium channel antagonist nifedipine affects endogenous Wnt5a levels at glutamatergic synapses in cultured hippocampal neurons (Bian et al., 2015; McLeod et al., 2018). These results demonstrate that changes in neuronal activity regulate the expression and/or release of certain Wnt proteins in hippocampal neurons. Given the increased levels of Wnt7a/b at dendritic spines, these findings suggest that the levels of Wnt proteins can be regulated locally.

**Figure 3:**
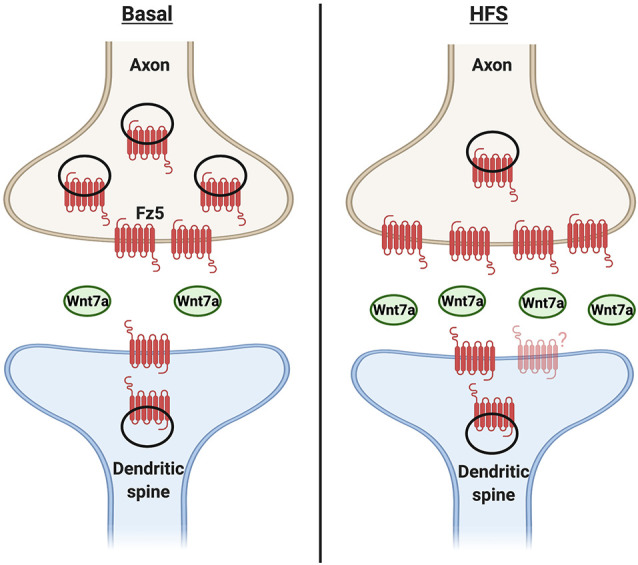
Cooperativity between neuronal activity and Wnt7a signaling in synapse formation. Under basal conditions, Wnt7a is present at the synapse. A fraction of Frizzled 5 (Fz5) receptor is also localized to both sides of the synapse, with relatively moreof the receptor at the presynaptic side. Changes in neuronal activity such as high-frequency stimulation (HFS) increase the expression and/or release of Wnt7a. In turn, Wnt7a increases the proportion of synaptic surface Fz5, thus changing the magnitude of Wnt signaling and contributing to activity-mediated synapse formation. However, it is unknown if the levels or localization of Fz5 at the postsynaptic side are modulated by neuronal activity.

Changes in neuronal activity also regulate the localization of Wnt receptors to the cell surface and particularly at the synapse (Figure 3). Surface biotinylation experiments showed that potassium chloride depolarization increases the surface expression of Fz5 (sFz5; Sahores et al., 2010). Although Fz5 receptors are localized to both sides of the synapse, they are not enriched at dendritic spines, suggesting the increase in sFz5 is primarily presynaptic (McLeod et al., 2018). This finding led to further investigation of the role of neuronal activity in the localization of Fz receptors to the synapse. Interestingly, different patterns of neuronal activity elicit different changes in synaptic sFz5. In cultured hippocampal neurons, HFS increases sFz5 and mobilization of sFz5 to the synapse, whilst low- frequency stimulation decreases both sFz5 and its synaptic localization (Sahores et al., 2010). The total levels of Fz5 protein are unchanged after either pattern of stimulation, suggesting that changes in neuronal activity mainly affect the trafficking of this Wnt receptor to the cell surface and its localization to the synapse.

What are the mechanisms through which HFS increases synaptic sFz5? Given that neuronal activity regulates Wnt expression and/or release, Sahores et al. (2010) tested the idea that the increase in synaptic sFz5 following HFS might be dependent on endogenous Wnt ligands. Consistent with this hypothesis, the HFS-mediated increase in synaptic sFz5 is attenuated by exposure to both sFRPs and Fz5 CRD. Thus, the trafficking of sFz5 to the synapse requires endogenous Wnts that bind to Fz5. Importantly, Fz5 blockade also abolishes HFS-mediated synapse formation (Sahores et al., 2010). In summary, these studies demonstrate that changes in neuronal activity such as HFS regulate Wnt signaling by modulating the expression and/or release of Wnt ligands such as Wnt7a/b and also by promoting the synaptic localization of Fz5. Thus, the increased localization of Wnt ligands and their receptors at the synapse contribute to activity-mediated synapse formation.

## Discussion

Here we discussed the role of Wnt signaling in the formation of excitatory synapses during development and in response to neuronal activity. In the adult hippocampus, growing evidence suggests that neuronal activity and Wnt signaling cooperate to promote synapse remodeling and synaptic plasticity. Changes in neuronal activity in hippocampal neurons modulate the expression and/or release of Wnts such as Wnt7a/b, which in turn affects the synaptic localization of Wnt receptors. Collectively, changes in the synaptic levels of Wnts and their receptors affect the magnitude of Wnt signaling, contributing to activity-mediated synapse formation.

**Figure 4:**
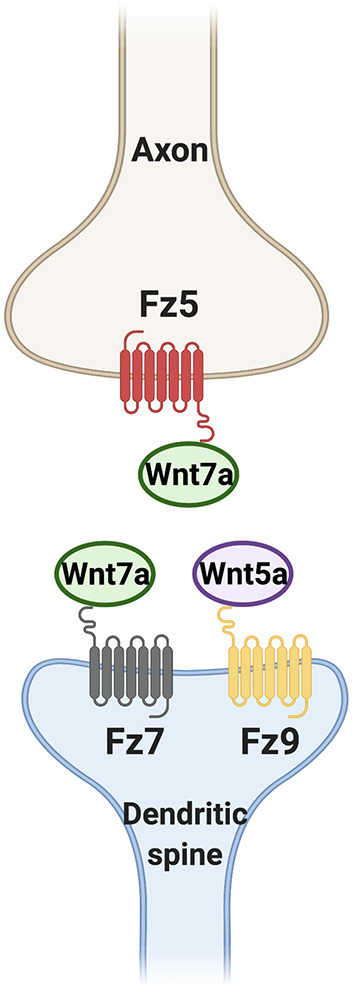
Wnt ligands promote synapse formation through different Fz receptors. Wnt7a signaling through Frizzled 5 (Fz5) and Fz7 is required for presynaptic assembly and dendritic spine formation respectively. Wnt5a through Fz9 is also sufficient but not required for the formation of dendritic spines.

During development, the assembly of synapses at both the presynaptic and postsynaptic sides require the activation of Wnt signaling pathways. Wnt7a and Wnt5a in particular have critical roles in this process (Figure 4). Wnt7a-Fz5 and Wnt7a-Fz7 signaling are necessary for presynaptic and postsynaptic assembly respectively. In contrast, Wnt5a specifically regulates postsynaptic assembly through Fz9 but it is not required for dendritic spine formation. Further studies are required to determine the mechanisms by which neuronal activity and Wnt signaling cooperate to promote synapse formation during postnatal development.

In addition to Wnts, a number of secreted and membrane-bound proteins also regulate synapse formation during development (Shen and Cowan, 2010; Südhof, 2018; Short et al., 2021). However, the cooperation between these different synaptogenic proteins is not fully understood. It is unclear if the different synaptogenic proteins act in a chronological manner or if they cooperate with each other at different stages of synapse assembly. Further studies in this area will provide a better understanding of how synapses are assembled.

In mammals, the cooperation between neuronal activity and Wnt signaling to promote synapse formation has been studied primarily in the hippocampus. Does such cooperativity also exist in other regions of the nervous system? Studies using non-mammalian model organisms could also provide further insight into this question. For example, retinotectal synapses in the developing *Xenopus* optic tectum undergo synaptic remodeling in response to repetitive exposure to unidirectional moving visual stimuli (Engert et al., 2002). Interestingly, exposure to the Wnt antagonist sFRP-2 markedly reduces synaptic remodeling in the dorsal tectum after exposure to visual stimuli. These results demonstrate that Wnt signaling is required for activity-mediated synaptic remodeling in the *Xenopus* dorsal tectum (Lim et al., 2010). Future studies will shed new light not only on how cooperativity between neuronal activity and Wnt signaling regulates synapse formation but also on their role in synaptic remodeling and synaptic plasticity. These studies will provide new avenues to restore neuronal connectivity in neurodevelopmental and neurodegenerative disorders.

## Author Contributions

ST performed the literature search, made the outline, and wrote the review. PS provided input on the content of the review and critically evaluated its content and contributed to the writing. All authors contributed to the article and approved the submitted version.

## Conflict of Interest

The authors declare that the research was conducted in the absence of any commercial or financial relationships that could be construed as a potential conflict of interest.
